# The Effect of Human and Bovine Milk Osteopontin on Intestinal Caco-2 Cells: A Transcriptome Comparison

**DOI:** 10.3390/nu15051166

**Published:** 2023-02-25

**Authors:** Brian Christensen, Albert J. Buitenhuis, Lotte N. Jacobsen, Marie S. Ostenfeld, Esben S. Sørensen

**Affiliations:** 1 Department of Molecular Biology and Genetics, Aarhus University, DK-8000 Aarhus, Denmark; 2 Center for Quantitative Genetics and Genomics, Aarhus University, DK-8000 Aarhus, Denmark; 3 Arla Foods Ingredients Group P/S, DK-8260 Viby J, Denmark

**Keywords:** transcriptome, Caco-2 cell line, human, bovine, osteopontin, gastrointestinal digestion

## Abstract

Osteopontin (OPN) is a multifunctional protein abundantly present in human milk, whereas the concentration is significantly lower in bovine milk. Human and bovine milk OPN are structurally similar and both proteins resist gastric digestion and reach the intestines in a bioactive form. Intervention studies have indicated the beneficial effects of supplementing infant formula with bovine milk OPN and several in vivo and in vitro studies have shown that bovine milk OPN positively influences intestinal development. To investigate the functional relationship, we compared the effect of simulated gastrointestinal digested human and bovine milk OPN on gene expression in Caco-2 cells. After incubation, total RNA was extracted and sequenced and transcripts were mapped to the human genome. Human and bovine milk OPN regulated the expression of 239 and 322 genes, respectively. A total of 131 genes were similarly regulated by the OPNs. As a control, a whey protein fraction with a high content of alpha-lactalbumin had a very limited transcriptional impact on the cells. Enrichment data analysis showed that biological processes related to the ubiquitin system, DNA binding, and genes associated with transcription and transcription control pathways were affected by the OPNs. Collectively, this study shows that human and bovine milk OPN have a significant and highly comparable effect on the intestinal transcriptome.

## 1. Introduction

Thousands of years of evolution have optimized mammals’ milk to provide their offspring with the optimal nutritive solution. Milk contains components that provide the neonate with both the necessary caloric energy and the building blocks needed for growth and development. Furthermore, milk contains numerous bioactive components, which provide signals and activities beyond classical nutrition. The majority of these bioactive components are proteins and encrypted peptides released during digestive processes. Human breast milk has been shown to contain proteins and peptides that possess antimicrobial activities, or play roles in the development of the gut and immune system [1], e.g., lactoferrin and alpha-lactalbumin which both are considered supplements for infant formula to reduce the well-known differences in gut development and maturation of immune responses between breastfed and infant formula-fed infants [2,3,4].

Another milk protein that has gained interest for its bioactivities is osteopontin (OPN). OPN is an acidic glycosylated and highly phosphorylated protein involved in a wide range of physiological processes including immunomodulatory activities, regulation of biomineralization, and brain and gut development [5,6]. OPN is an intrinsically disordered protein that contains several integrin-binding motifs through which it initiates signaling cascades by binding to cell receptors.

OPN is encoded by a single gene but is expressed in many different tissues and body fluids such as urine, blood, and most abundantly in milk. Milk from Danish mothers contains 99.7 to 138 mg OPN per liter, whereas the content in milk from Asian mothers is significantly higher, at 185.0 to 266.2 mg/L [7,8]. In contrast, bovine milk and infant formula only contain 18 and 9 mg/L, respectively [7]. This deficit has led to the development of infant formulas enriched with bovine milk OPN to close the concentration gap to human milk. The high concentration of OPN in human milk, cord blood, and infant plasma [7] suggests that OPN plays a role in infant growth and development. Human and bovine milk OPN are resistant to proteolysis by neonatal gastric aspirates [9] and an OPN fragment containing the functional integrin-binding motifs is protected from gastric digestion by *O*-glycosylated residues in the protein [10]. Thus, it is likely that part of the ingested OPN resists digestion during the passage through the stomach, and is able to bind receptors at the intestinal surfaces. The amino acid sequences of bovine and human OPN are highly homologous with 182 identical amino acids (61%) and another 44 residues are structurally conserved substitutions. Furthermore, integrin binding motifs and sites of phosphorylation, glycosylation, and regulatory proteolytic cleavage are conserved between the two species [11]. Consequently, bovine and human milk OPN are likely to be able to substitute for each other in physiological processes in the intestine.

Human and bovine milk OPN can stimulate the proliferation of human crypt-like intestinal cells in vitro, and human OPN stimulates intestinal immunity by upregulation of IL-18 expression [12,13]. OPN is a key cytokine in the regulation of the Th1/Th2 balanced immune response and bovine milk OPN can induce the expression of IL-12 from human intestinal lamina propria mononuclear cells [7,14]. In a rodent study, OPN-knockout suckling mice were more susceptible to rotavirus infection and showed more intense and prolonged diarrhea than wild-type suckling mice [15]. Likewise, administration of bovine milk OPN in the drinking water had a protective effect in a mouse model of colitis [16], and OPN-knockout dams supplemented with bovine milk promoted beneficial effects on intestinal growth measured as villus height and crypt depth after LPS challenge [6]. Similarly, a milk diet supplemented with bovine milk OPN in a preterm pig model improved the villus-to-crypt ratio [17].

In a clinical trial, infants fed formula supplemented with bovine milk OPN had reduced levels of the pro-inflammatory cytokine TNF-α and fewer days with fever compared to infants receiving non-supplemented formula [18]. In a study with 3-month-old rhesus monkeys, the addition of OPN to formula significantly shifted the intestinal gene expression toward that of breastfed infant monkeys [19]. Collectively, these studies suggest that bovine milk OPN can affect intestinal development during early life, and recently, bovine OPN has received a positive opinion for use as a novel food ingredient in infant nutrition by the European Food Safety Authorities [20].

In the present study, we used human colon adenocarcinoma (Caco-2) cell monolayers as an in vitro model of the intestinal epithelium and investigated how the cells responded to treatment with simulated gastrointestinal digested OPN by gene expression analysis using RNAseq. We identified 239 and 322 differentially expressed genes (DEGs) between control cells and cells treated with human and bovine milk OPN, respectively, with a strong correlation between the two treatments. In contrast, a digest of a whey protein fraction with a high content of alpha-lactalbumin (ALA) had a very limited transcriptional impact on the Caco-2 cells.

## 2. Materials and Methods

### 2.1. Proteins

The whey protein fraction Lacprodan**^®^** Alpha-20 with a minimum of 57% alpha-lactalbumin (ALA) and bovine milk OPN (Lacprodan**^®^** OPN-10) was obtained from Arla Foods Ingredients, Viby, Denmark. Lacprodan**^®^** OPN-10 contains ~78% protein, of which 95% is OPN. Human OPN from breast milk was purified by anion-exchange chromatography on DEAE-Sepharose CL-6B (GE Healthcare) and barium chloride and sodium citrate precipitation as described [21].

### 2.2. In Vitro Simulation of Gastrointestinal Digestion

The proteins were subjected to digestion essentially as described [22,23]. The proteins were dissolved in 0.15 M NaCl, pH 2.5 at a concentration of 1 mg/mL and incubated with pepsin from porcine gastric mucosa (Merck) in a 1:50 *w*/*w* enzyme-to-substrate ratio for 60 min at 37 °C. Before digestion with pancreatic proteases, the samples were lyophilized and washed twice in deionized water. Trypsin and chymotrypsin from bovine pancreas (Worthington Biochemical Corporation) (1:50 *w*/*w*) and elastase (Worthington) (1:250 *w*/*w*) were added in 50 mM ammonium bicarbonate buffer and the mixture was incubated for 60 min at 37 °C followed by inactivation of the proteases by addition of phenylmethanesulfonyl fluoride to a concentration of 1.5 mM.

### 2.3. Cells

The human colorectal adenocarcinoma cell line, Caco-2 was obtained from DSMZ (Braunschweig). The cells were grown to 50–60% confluence in Dulbecco’s modified Eagle’s medium with Glutamax cell culture medium supplemented with 10% heat-inactivated fetal bovine serum, 100 units/mL penicillin, and 100 μg/mL streptomycin (all from Invitrogen) in a humidified 5% CO_2_/95% air atmosphere at 37 °C. The cells used in the experiments were between passages 40 and 50.

The Caco-2 cells were seeded in 24-well tissue culture-treated polystyrene plates (Corning Glass) (150,000 cells/well). After differentiation for 21 days, the cells were washed twice in phosphate-buffered saline (PBS), and incubated with digested proteins (6.2 µM) in a serum-free medium at 37 °C for 2 h (*n* = 4). As a control, cells were incubated with proteases and phenylmethanesulfonyl fluoride in concentrations identical to the concentration used in the digestion of the proteins. Then, the cells were washed twice with PBS, trypsinized, and centrifuged for 5 min at 5000× *g* at 4 °C. The cell pellets were further washed twice with PBS and centrifuged for 5 min at 5000× *g* at 4 °C. The pellet was stored at −80 °C until RNA purification.

### 2.4. RNA Purification, Library Preparation, and Sequencing

These services were supplied by Omiics Aps (Aarhus, Denmark). Total RNA was purified using trizol per the manufacturer’s instructions. Purified RNA was quality controlled by an Agilent Bioanalyzer 2100 using the RNA 6000 Nano assay. The Lexogen QuantSeq 3′ mRNA-Seq Library Prep Kit FWD was used for the preparation of sequencing libraries. Quality control of the libraries was performed using the Agilent Bioanalyzer 2100 High Sensitivity DNA Assay and quantified by quantitative real-time PCR. Libraries were pooled according to library concentration and sequenced as 50 bp single end on an Illumina HiSeq 4000 sequencer. Sequencing data were quality filtered using FastX-Toolkit requiring a quality score of 30 on at least 80% of sequencing reads, and adapter removal was performed using Cutadapt [24].

### 2.5. Mapping of the Sequences to the Genome

Tophat was used to map the filtered RNA-seq data to the human genome (hg19) [25]. The number of reads mapping to RefSeq annotated genes were counted using bedtools [26]. The file provided by the lab contained the basic gene information: chromosomal position and gene name. To extract additional information such as gene description and Gene Ontology (GO) annotation, the R library BioMart (version 2.52.0) was used (https://grch37.ensembl.org/info/data/biomart/index.html) (accessed on 15 October 2019).

### 2.6. Differential Expressed Genes

The count data of the RNA sequences were read using R (version 3.6.1; https://www.r-project.org/) (accessed on 15 October 2019). The DEGs were determined for three contrasts: human milk OPN vs control; bovine milk OPN vs control; and ALA vs control using the R library DESeq2 (https://bioconductor.org/packages/release/bioc/html/DESeq2.html) (accessed on 15 October 2019). The overlapping genes between the three treatments were shown using a Venn diagram [27] using the FDR threshold [28] of *p*-adjust < 0.05 and a log_2_|FC| ≥ 1. DEGs regulated by human and bovine milk OPN (*p*-adjust < 0.05 and a log_2_|FC| ≥ 1) were subjected to Gene Ontology GO and Kyoto encyclopedia of genes and genomes (KEGG) pathway analyses to identify genes with similar functions using the Database for Annotation, Visualization and Integrated Discovery (DAVID; https://david.ncifcrf.gov/) (accessed on 17 June 2022) [29]. To improve the accuracy and credibility of the identified DEGs for strict comparison, *p*-adjust < 0.01 and a log2|FC| ≥ 1 were used as thresholds. Only database information from cells of epithelial origin is included.

## 3. Results

The total number of unique transcripts obtained was 170,492 with an average length of 1010.8 bp. All transcripts were assigned to the GRCh37 genome build identifying 19,443 unique genes (Table 1).

**Figure 1:**
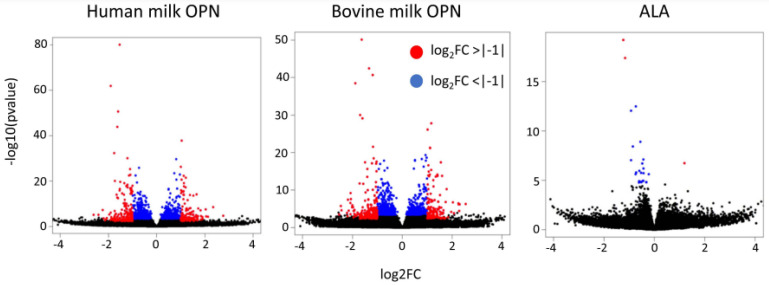
Volcano plots of the human milk OPN, bovine milk OPN, and a whey protein fraction with a high content of alpha-lactalbumin (ALA) treatments. Red (log_2_FoldChange >|−1|) and blue (log_2_FoldChange <|−1|) dots show the significant genes.

A total of 239 and 322 DEGs were significantly regulated in Caco-2 cells by human and bovine milk OPN, respectively (Table S1). In contrast, only five DEGs were identified in the ALA group compared to the control (Table S1). The gene distributions are visualized in a Volcano plot (Figure 1), where the DEGs at the left and right sides of the Volcano plot showed no apparent differences in the proportion of upregulated and downregulated genes for the OPN treatments.

**Figure 2:**
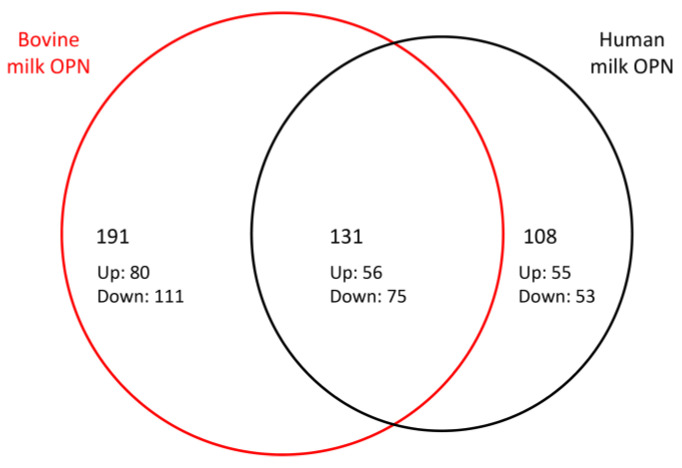
Differentially expressed genes in Caco-2 cells exposed to bovine milk OPN and human milk OPN. The Venn diagram shows the shared and OPN-specific genes. *p*-adjust <0.05 LCF > |1|.

A Venn diagram was constructed to show the relationship between DEGs regulated by human and bovine milk OPN (Figure 2). Among the 239 and 322 DEGs in the human and bovine OPN groups, 131 DEGs were observed after both treatments, showing that 55% of the genes modulated by human OPN were also modulated by bovine OPN. Among the 131 shared DEGs, 56 were upregulated (43%) and 75 genes were downregulated (57%) and except for a single gene (NEDD4), all of the 131 shared DEGs were similarly up- or downregulated by human and bovine milk OPN, respectively (Table S1). When analyzing the twenty most up- and downregulated DEGs after treatment with human milk OPN using more stringent thresholds (P-adj < 0.01, and |log2FC| > 1), the similarity of the human and bovine OPN groups increases to 75% (Table 2 and Table 3).

In order to search for significantly enriched gene ontology classes, the DEGs were used to identify enriched GO categories and KEGG pathways using the DAVID bioinformatics program. The results of the GO analysis of the human milk OPN DEGs were significantly enriched in categories related to ‘protein ubiquitination’ and ‘regulation of transcription from RNA polymerase II’ (Table 4). The DEGs modulated by bovine milk OPN treatment of Caco-2 cells were also related to ‘regulation of transcription from RNA polymerase II’ as well as ‘DNA helicase activity’, ‘DNA binding’, and ‘Cysteine-type deubiquitinase activity’. Even though some GO annotations were only significantly enriched in one of the OPN groups, DEGs were observed from both the human OPN and bovine OPN treatment of the Caco-2 cells in all identified GO annotations (Table 4). The individual DEGs enriched in the different biological GO processes are listed in Supplementary Table S2. The KEGG analysis indicated that the pathways ‘mRNA surveillance pathway’ and ‘spliceosome’ were enriched in the DEGs of the human milk OPN-treated Caco-2 cells (Table 4). The groups of observed GO/KEGG categories contain genes among the 20 most up- and downregulated DEGs (Table 2 and Table 3), such as PCF11, CHD1, MAFF, ZSCAN26, JADE1, PRDM1, ZNF239 (in ‘regulation of transcription from RNA polymerase II’ and ‘DNA binding’), and SMURF1 (in ‘Protein ubiquitination’).

## 4. Discussion

Human milk contains ~100–250 mg/L (~3–7.5 µM) OPN which corresponds to up to ~2.1% (*w*/*w*) of the total protein content, whereas bovine milk and infant formula contain only 18 mg/L and 9 mg/L, respectively [7,8]. Bovine and human milk OPN are structurally very similar and both are resistant to gastrointestinal digestion [10,11]. Safety evaluations have shown that bovine milk OPN is not genotoxic in mice, rats, and human cell lines [30] and it shows no adverse effects in infant rhesus monkeys or in human infants [18,19]. Recently, bovine OPN has received a positive opinion for use in infant nutrition products by EFSA [20].

In this study, we used the established Caco-2 cell monolayer system to compare the effects of human and bovine milk OPN on intestinal enterocytes. Caco-2 cells are widely used as in vitro models of the intestinal epithelium as they spontaneously differentiate in culture to form a confluent monolayer of polarized cells showing structural and functional characteristics similar to mature enterocytes. In particular, differentiated Caco-2 monolayers display microvillus structure, carrier-mediated transport systems, and tight junctions [31,32]. We have previously shown that factors such as apparent permeability, transendothelial electrical resistance, and expression of tight junction proteins indicate that the cells successfully form a monolayer functioning in vitro as a model of the intestinal epithelium [33].

RNA sequencing of Caco-2 cells after stimulation with digested OPN in a concentration similar to that found in milk showed that 239 and 322 genes were significantly regulated by human and bovine milk OPN, respectively. Of these DEGs, 131 genes were similarly up- or downregulated by both OPNs corresponding to ~55% of the DEGs modulated by human milk OPN. The effects on gene expression were even more identical among the twenty most up- and downregulated DEGs, where ~75% of genes were shared (Table 2 and Table 3). This shows that human OPN and bovine milk OPN regulate gene transcription in the Caco-2 model of the intestinal epithelium in a very similar manner. In comparison, only five DEGs were identified after treatment of the Caco-2 cells with a digest of a whey protein fraction with a high content of alpha-lactalbumin. OPN binds to the α_V_β_1_, α_5_β_1,_ α_V_β_5,_ α_V_β_6,_ and α_V_β_3_-integrins via the conserved Arg-Gly-Asp (RGD) sequence, whereas the α_4_β_1,_ and α_9_β_1_-integrins interact with a non-RGD motif, SVVYGLR, in OPN [5]. Caco-2 cells express several of these integrins including α_4_β_1_, α_V_β_1_, and α_V_β_3_ [34]_,_ which potentially could participate in the signaling mediated by OPN.

The α-arrestin domain-containing protein-3 (ARRDC3) was the most upregulated DEG in response to human OPN (Table 2). ARRDC3 is a member of the arrestin superfamily and has been shown to suppress metastatic breast cancer by inducing ubiquitination and degradation of the β_2_-adrenergic receptor and the β_4_-integrin [35,36]. ARRDC3 also regulates insulin receptor signaling in the liver by directly interacting with the insulin receptor and thereby ARRDC3 could play roles in hepatic suppression of gluconeogenesis [37].

In a study, comparing the effects of lactoferrin from human and bovine milk on intestinal transcriptomic profiling using human intestinal epithelial crypt-like cells, it was shown that 150 and 395 DEGs were significantly regulated by human and bovine lactoferrin, respectively [38]. Of these, 29 DEGs were regulated by both human and bovine lactoferrin showing a rather low similarity in the gene expression. In the present study, 55% of the genes regulated by human OPN were also regulated by bovine OPN, which indicates that the replacement of human OPN for bovine OPN is more conservative with regard to the influence on gene expression than the replacement of human for bovine lactoferrin. This is further substantiated in a previous study in which 1017 intestinal genes were differently expressed between formula-fed and breastfed rhesus infants, but the addition of bovine milk OPN to the formula reduced the difference to only 217 genes [19]. Among the genes regulated by OPN in the rhesus monkey transcriptome, 14 and 26 DEGs were regulated by human milk OPN and by bovine milk OPN, respectively, in our study. Several of these genes are involved in actin binding or transcriptional processes (Table S3). In addition, 17 genes from the rhesus monkey study were associated with protein ubiquitination, which corresponds well with the GO information of genes in the present study that linked 25 and 40 human and bovine OPN DEGs, respectively, to ubiquitination (Table 4 and Table S2). Previously, OPN has been shown to potentially affect innate antiviral immunity by inhibition of poly-ubiquitination and degradation of the cytoplasmic signaling protein TRAF3 [39]. Molecular function ontology reports of the rhesus infant study also showed that GO pathways such as ‘mRNA splicing via spliceosome’, ‘Transcription–mRNA processing’, and ‘Transcription by RNA polymerase II’ were influenced by OPN [19]. Likewise, in the present study GO and KEGG analyses indicated that human milk OPN primarily modulated genes associated with ‘protein ubiquitination’ and ‘regulation of transcription from RNA polymerase II’. Otherwise, ‘DNA Binding’, ‘DNA helicase activity’, and ‘Cysteine-type deubiquitinase activity’ were among the processes correlated with exposure of the Caco-2 cells to bovine milk OPN. Several DEGs from both the human OPN and bovine OPN treatments were observed in most of these biological processes suggesting a rather similar response of the intestinal cells to the OPNs (Table 4). The KEGG pathway result revealed that human milk OPN modulated DEGs involved in ‘mRNA surveillance pathway’ and ‘spliceosome’. Collectively, these results and the upregulation of ARRDC3 suggest that the OPNs play a role at the transcriptional level and in the regulation of intracellular pathways through the ubiquitination system. However, these molecular functions are solely based on gene expression data and further studies are needed to conclude that the transcripts result in functional proteins.

Interestingly, the gene for ILF-2 (interleukin enhancer-binding factor 2) was upregulated in response to both human milk OPN (Fold change 2.04) and bovine milk OPN (Fold change 2.23) (Supplementary Table S1), and ILF-2 was among the DEGs enriched in the GO term ‘DNA binding’ for both OPNs (Table S2). ILF-2 is a transcription factor required for T-cell expression of interleukin 2 [40], which is of interest as interleukin-2 is a cytokine involved in oral tolerance and immunity through direct interactions with T-cells [41]. Thus, our data are in line with a randomized controlled trial showing that human infants fed formula supplemented with bovine milk OPN had similar plasma levels of interleukin-2 as breastfed infants. Moreover, these levels were higher than observed in infants fed regular formula [18]. Collectively, the upregulation of ILF-2 substantiates that OPN can affect the expression of interleukin-2 from T cells.

## 5. Conclusions

OPN is a multifunctional protein expressed in many tissues and body fluids, with the highest concentrations in milk. The results of the present study show that gastrointestinal digested OPN from human and bovine milk has a significant and comparable effect on the gene expression of intestinal Caco-2 cells. The human and bovine OPN digests regulated 239 and 322 genes, respectively, and of these 131 genes were shared by both proteins. In contrast, an alpha-lactalbumin-rich whey fraction only modulated the expression of five genes. The differently expressed genes affected biological processes related to the ubiquitin system, DNA binding, and genes associated with transcription and transcription control pathways. These data further indicate that bovine milk OPN and human milk OPN deliver similar signals at the intestines, which are responsible for the physiological effects of orally ingested OPN previously reported in both animal studies and human interventions. Furthermore, it highlights the potential applications of bovine milk OPN as a novel food ingredient in infant nutrition. As this is an in vitro study its results must be verified by in vivo studies before clear conclusions about the effect of OPN can be drawn.

## Figures and Tables

**Table 1:** Summary of the RNA-sequence dataset of the Caco-2 cell line.

Type	Resource
No. of unique transcripts	170,492
Maximum transcript length	482,375 bp
Minimum transcript length	16 bp
Median transcript length	70 bp
Average transcript length	1010.8 bp
No. unique genes	19,443

**Table 2:** Twenty most upregulated differentially expressed genes (by Fold change)(*p*-adjust <0.01 LCF > |1|). Human milk OPN (hOPN) and bovine milk OPN (bOPN).

Symbol	Fold Change		Molecular Function
	hOPN	bOPN	
STPG3-AS1	6.7	-	Not available
ARRDC3	5.1	2.7	Beta-3 adrenergic receptor binding (GO:0031699)
ZSCAN26	4.4	-	DNA-binding transcription factor activity (GO:0000981)
MUC19	4.1	2.9	Gel-forming mucin protein family
JADE1	4.1	2.8	Transcription co-activator activity (GO:0003713)
TMEM267	3.6	5.8	Protein binding (GO:0005515)
NDRG1	3.6	3.1	Small GTPase binding (GO:0031267)
INTS5	3.3	3.4	Protein binding (GO:0005515)
WDR70	3.3	4.1	Enzyme binding (GO:0019899)
WDR53	3.2	4.8	Protein binding (GO:0005515)
MTFMT	3.1	3.0	Methionyl-tRNA formyltransferase activity (GO:0004479)
APOL6	3.1	-	Lipid binding (GO:0008289)
NOS2	2.95	2.6	Nitric-oxide synthase activity (GO:0004517)
DGKZ	2.9	2.8	ATP binding (GO:0005524)
RBM14	2.86	2.92	RNA binding (GO:0003723)
ARHGEF11	2.85	2.70	G protein-coupled receptor binding (GO:0001664)
TYMS	2.80	2.18	Thymidylate synthase activity (GO:0004799)
PRDM1	2.71	-	DNA-binding transcription repressor activity (GO:0001227)
GAL	2.64	2.37	Galanin receptor activity (GO:0004966)
ZNF239	2.63	2.40	DNA binding (GO:0003677)

**Table 3:** Twenty most downregulated differentially expressed genes (by Fold change)(*p*-adjust <0.01 LCF > |1|). Human milk OPN (hOPN) and bovine milk OPN (bOPN).

Symbol	Fold Change		Molecular Function
	hOPN	bOPN	
BMP2	0.16	0.22	Protein serine/threonine kinase activator activity (GO:0043539)
HSPB8	0.19	0.18	Protein homodimerization activity (GO:0042803)
IPO9	0.24	0.27	Small GTPase binding (GO:0031267)
AP5B1	0.24	-	Protein binding (GO:0005515)
PCF11	0.25	0.25	RNA polymerase II complex binding (GO:0000993)
MAPKBP1	0.27	0.32	Protein binding (GO:0005515)
PPP1R15A	0.30	0.35	Protein phosphatase regulator activity (GO:0019888)
RASA4DP	0.30	0.24	GTPase activator activity (GO:0005096)
DIDO1	0.30	0.22	RNA binding (GO:0003723)
SMURF1	0.30	0.37	Ubiquitin-protein transferase activity (GO:0004842)
SC5D	0.30	0.40	Sterol desaturase activity (GO:0000248)
PINK1-AS	0.31	-	Not available
CYCS	0.31	-	Electron transfer activity (GO:0009055)
PCYOX1	0.32	0.24	mRNA binding (GO:0003729)
RTKN2	0.33	0.40	Positive regulation of NIK/NF-kappaB signalling (GO:1901224)
CHD1	0.34	0.38	DNA binding (GO:0003677)
MAFF	0.34	0.38	DNA-binding transcription factor activity (GO:0000981)
SYNE2	0.34	-	Positive regulation of cell migration (GO:0030335)
LDLR	0.35	0.39	Low-density lipoprotein particle binding (GO:0030169)
LINC01277	0.36	0.45	Not available

**Table 4:** Gene Ontology and KEGG pathway analysis of DEGs associated with Caco-2 cells after OPN treatment.

Category	Term/Gene Function	Gene Count		*p*-Value	
		hOPN	bOPN	hOPN	bOPN
GO:0016567	Protein ubiquitination	11	13	0.037	N.S.
GO:0006357	Regulation of transcription from RNA polymerase II	35	40	0.00024	0.0028
GO:0003678	DNA helicase activity	1	5	N.S.	0.0135
GO:0003677	DNA binding	27	34	N.S.	0.0018
GO:0004843	Cysteine-type deubiquitinase activity	4	6	N.S.	0.034
hassa03015	mRNA surveillance pathway	5	3	0.01	Nhas
hsa03040	Spliceosome	5	1	0.044	N.S.

N.S.: Not significant.

## Data Availability

Not applicable.

## Supplementary Materials

The following supporting information can be downloaded at: https://www.mdpi.com/article/10.3390/nu15051166/s1, Table S1: Differentially expressed genes (DEGs) in the Caco-2 cells in response to human milk OPN, bovine milk OPN, and ALA. Table S2: Gene Ontology (GO) and KEGG analysis for the DEGs in the Caco-2 cells, upon treatment with human milk OPN or bovine milk OPN. Table S3. DEGs observed in the present study and in Donovan et al. 2014. [19].
